# ‘Nothing can be done until everything is done’: the use of complexity arguments by food, beverage, alcohol and gambling industries

**DOI:** 10.1136/jech-2017-209710

**Published:** 2017-10-04

**Authors:** Mark Petticrew, Srinivasa Vittal Katikireddi, Cécile Knai, Rebecca Cassidy, Nason Maani Hessari, James Thomas, Heide Weishaar

**Affiliations:** 1 Faculty of Public Health and Policy, London School of Hygiene and Tropical Medicine, London, UK; 2 MRC/CSO Social and Public Health Sciences Unit, University of Glasgow, Glasgow, UK; 3 Department of Anthropology, Goldsmiths University of London, London, UK; 4 EPPI-Centre, SSRU, Department of Social Science, UCL Institute of Education, University College London, London, UK; 5 Hertie School of Governance, Berlin, Germany

**Keywords:** alcohol, epidemiology, obesity, public health policy

## Abstract

**Background:**

Corporations use a range of strategies to dispute their role in causing public health harms and to limit the scope of effective public health interventions. This is well documented in relation to the activities of the tobacco industry, but research on other industries is less well developed. We therefore analysed public statements and documents from four unhealthy commodity industries to investigate whether and how they used arguments about complexity in this way.

**Methods:**

We analysed alcohol, food, soda and gambling industry documents and websites and minutes of reports of relevant health select committees, using standard document analysis methods.

**Results:**

Two main framings were identified: (i) these industries argue that aetiology is complex, so individual products cannot be blamed; and (ii) they argue that population health measures are ‘too simple’ to address complex public health problems. However, in this second framing, there are inherent contradictions in how industry used ‘complexity’, as their alternative solutions are generally not, in themselves, complex.

**Conclusion:**

The concept of complexity, as commonly used in public health, is also widely employed by unhealthy commodity industries to influence how the public and policymakers understand health issues. It is frequently used in response to policy announcements and in response to new scientific evidence (particularly evidence on obesity and alcohol harms). The arguments and language may reflect the existence of a cross-industry ‘playbook’, whose use results in the undermining of effective public health policies – in particular the undermining of effective regulation of profitable industry activities that are harmful to the public’s health.

## Introduction

Unhealthy commodity industries (UCIs) are responsible for producing and promoting the unhealthy consumption of products, which play a major role in driving the growing burden of non-communicable diseases.^1^ The strategies that such industries use to defend their practices are increasingly the subject of research.^1–3^ There is growing evidence that these activities, on the part of the tobacco and alcohol industry at any rate, have involved the misuse and misrepresentation of the scientific process and the evidence base, sometimes through the adoption of ‘pseudo-scientific’ processes and language.^4 5^ Drawing on apparently scientific concepts and methods in this way has the goal of changing how policy issues are understood and debated. It also tends to manufacture uncertainty and undermine scientific consensus, thereby curtailing the potential for effective public health policy responses.^1^ Such discourses can exert an impact on the real world of policymaking. For example, the tobacco industry fostered the use of the concepts of psychological stress as an alternative explanation for coronary heart disease (CHD), sponsoring researchers and conferences and using the concepts in litigation to argue that these acted as unmeasured confounders in the relationship between smoking and disease.^6 7^ Other UCIs pursue similar tactics.^8–13^ However, comparisons of discourses across UCIs remain uncommon.

One candidate for further analysis is complexity. Complexity science is increasingly important in public health,^14–17^ stimulated by a recognition that many public health problems arise in unpredictable ways from the interactions between individuals and their environments.^14 18 19^ For example, obesity is subject to influences ranging from the individual level (such as individual choices and energy needs) to the system level (eg, advertising, pricing, agricultural policies). In recognition of this, frameworks such as the Foresight model have been developed to illustrate this complexity and to identify intervention points.^20^ Other major public health challenges have also been subject to complexity analysis, including tobacco control^18^ and alcohol consumption and harms^14 21 22^ in order to show how the problems emerge from highly interconnected systems.^19 23^

In parallel, it is possible that UCIs may be using the concept of complexity to quite different ends: to dispute the role of unhealthy commodities in the causation of health problems and to prevent the adoption of evidence-based interventions, particularly those that regulate the environment in which the industry acts. While there has been a wealth of research on the activities of the tobacco industry, other key commercial actors that influence the disease burden—notably, alcohol, food and sugar-sweetened beverage (SSB) industries—are believed to be pursuing similar strategies.^24^ Research on these industries has recently expanded to include gambling, but it remains under-researched.^25^ Gambling bears similarities to alcohol and tobacco; for example, there are no safe levels of alcohol and tobacco use, and it has been argued that there are no safe levels of gambling.^26^

We aimed to investigate how four UCIs (alcohol, food, SSBs and gambling) have used the concept of complexity. By making systematic comparisons across these industries, we seek to understand whether they pursue common tactics and the implications of this for research, practice and policy (see box 1).^12 27^

## Methods

We used established document analysis approaches, informed by the principles of Critical Discourse Analysis.^28 29^ Through a focus on the use of language, this approach allows power relations (including the exertion of policy influence) to be made visible. We first identified documents, interviews, presentations or statements in which four industries (the alcohol industry, the SSB industry, the food industry and the gambling industry) presented the scientific idea of ‘complexity’. We assessed how the concept was presented, what the explicit argument being made was and whether there were inherent contradictions within the data. This involved a general thematic analysis to identify themes and subthemes.^30^

We identified examples from searching industry websites (the International Alliance for Responsible Drinking (IARD) and the International Center for Alcohol Policies (ICAP) and its publications; the Responsible Gambling Trust (RGT; rebranded as GambleAware in 2016); the Portman Group; Drinkaware; the UK Food and Drink Federation; the American Beverage Association), trade press (The Grocer and Just-Drinks.com) and reports of Health Select Committees. We included examples in which industry representatives refer to complexity; other examples from these industries are given in online supplementary file 1. The findings are then placed in a wider framework, to highlight how other industries have historically used the concept of complexity, and to identify general approaches.

10.1136/jech-2017-209710.supp1 Supplementary file 1

## Results

We identified two main themes in these documents:

### 1. The impossible complexity of public health problems

A recurrent theme within publicly available industry sources is the presentation of the drivers of public health issues as ‘complex’, often implying that the issue is influenced by a multitude of interconnected factors, many of which are outside of the control of policymakers and industry. Instead, the emphasis is usually placed on the responsibility of the individual. The industry in question generally does not include itself among the causal factors, but instead it often stresses the need to deal with the complexity via a multistakeholder, collaborative partnership approach. For example, the Portman Group, the UK alcohol industry’s corporate social responsibility body, appears to use complexity arguments to reject calls for alcohol labelling to be improved.^31^ Moreover, the alcohol industry frequently points to the many complex influences on young people’s drinking, in order to rebut arguments about the role of advertising and pricing, as in this example:

*“Young people’s drinking is influenced by a complex number of interacting factors including family, peers, media, cultural norms and government policies. Therefore, a complex range of solutions and the involvement of different stakeholders are needed to reduce the potential risk for harm.”*.^32^ ^32^

The World Spirits Alliance goes further, arguing in a submission to a WHO public hearing in 2009 on the WHO Global Strategy on Alcohol, that the complexity of the issue means that current evidence-based solutions are ineffective:

*“The reasons for alcohol misuse are complex, yet solutions are often simplistic, based around increased regulation… New solutions need to be found therefore. These must be multi-faceted and culturally sensitive. They should not be so wide as to try and reach the entire population… No one size fits all solution for tackling misuse of alcohol exists. There are wide cultural differences across, and even within, regions.”*^33^

Twenty of the 37 submissions from international spirits, wine and beer producers to the WHO Global Strategy on Alcohol referred to the complexity of the problem, and 25 argued against ‘one-size-fits-all’ solutions. The two concepts often appeared together, as in the following statement by the Brewers Association of Australia and New Zealand Inc. (2009):

*“Alcohol misuse is not a problem of simple, linear cause and effect relationships. Instead, a raft of complex and interacting factors underlie a society’s attitudes and actions towards alcohol use, dramatically reducing the likely effectiveness of any intervention taken in isolation. Therefore, it is essential that the background and diversity of societal and cultural settings be fully considered in any strategy”.*^33^

This quote also illustrates another aspect of complexity that is very often highlighted in these submissions and elsewhere as a reason why they claim that policies are likely to be ineffective: variations in cultural contexts.

An underpinning aspect of the use of complexity by these industries is the distancing of their role in contributing to the policy issue at hand. Such a framing may be chosen because it is of value in litigation. For example, McDonalds has successfully used arguments about the multifactorial causation of obesity to defend itself against a class action taken against it by two New York teenagers who argued that the company was liable for their obesity (Pelman vs McDonalds Corp, 2002).^34^ McDonalds argued successfully that obesity is complex and multifactorial^35^ and that the plaintiffs needed to show that other possible causes of their obesity (ie, other than a McDonalds diet) were ruled out.^36^ They argued:

*“The plaintiffs’… physical conditions are inherently the result of a combination of so many factors and influences that attempting to attribute proximate cause to the consumption of certain of the products served at McDonald’s is impossible as a matter of law.”*^37^

See also online supplementary file 1.

### 2. Rejecting evidence-based policies: ‘no simple solutions, no single ingredient’

Having argued for complexity in causation, industries then appear to use this as a platform from which to argue that simple solutions (often referring to regulation) would be ineffective. The international alcohol industry has long made extensive use of such arguments to confuse and create doubt about evidence on the causes of alcohol harms, while presenting alternative, usually ineffective, solutions. For example, the industry-funded body ICAP in its book ‘Drinking in Context’ (2007) argues that alcohol is such a complex behaviour that it is difficult to prove that interventions (typically, policies) really work.^38^ More recently, IARD’s message is that alcohol consumption is subject to so many cultural and contextual influences, that cause–effect relationships cannot be established and that interventions therefore cannot truly be ‘known’ to be effective.

The food industry uses very similar arguments, often claiming that because of the complexity of causation, it is impossible to single out one ingredient or product as a cause; such thinking is then describe as simplistic. For example, the director general of the Food and Drink Federation, the representative body for the UK food and drink manufacturing industry, in a 2015 letter to the head of the National Health Service in England, makes the case why sugar should not be blamed for obesity:

*“We believe obesity is a complex problem which cannot be reduced to the demonization of one ingredient… there is no simple answer to the complex problems of obesity”* (Food and Drink Federation (third June 2015)).^39^

SSB industry arguments, too, frequently contrast a ‘complex problem’ (obesity) with ‘simplistic approaches’ or ‘single solutions’ (typically, taxation of SSBs), as in this example:

*“There’s ample evidence to suggest that taxing soft drinks won’t curb obesity, not least because its causes are far more complex than this simplistic approach implies… Trying to blame one set of products is misguided…”* (British Soft Drinks Association (BDSA)^40^)

The gambling industry responds to challenges about its responsibility for harms in a similar way, rejecting industry-related causes of problem gambling, such as advertising or problem products.^41^ Typical examples include the industry’s endorsement of research funded by the RGT (now GambleAware), a charity funded by voluntary donations from the gambling industry. The research in question did not provide evidence deemed capable of supporting any changes to policy, including reductions in stakes or prizes:

*“Ladbrokes [UK-based betting company] welcomes this world-leading study which illustrates that gambling related harm is complex and that simplistic approaches are not effective.”*^42^

The idea that problematic gambling behaviour is complex and that existing research does not provide sufficient grounds for interventions is very widely employed and influential in the industry.^43 44^ It often appears to to identify the individual, rather than *any* products or activities, as the source of problem gambling. For example:

*Problem gambling is complex and is about the person not the specific product. Gala Coral, and the bookmaking sector as a whole, is determined to play a leadership role in identifying appropriate measures that improve player protection for those who need it while, as far as possible, protecting the freedom of the millions who enjoy betting responsibly.*^45^

The complexity argument is also used to reject changes to the structural characteristics of products such as Fixed Odds Betting Terminals, which are associated with high rates of problem gambling. This is seen in the Association of British Bookmaker’s (ABB) response to research by the Gambling Commission, which they suggested indicated that there was no relationship between gaming machines in bookmakers and problem gambling:

*This is yet another piece of evidence that shows problem gambling is complex and that focusing on stake or a single class of venue is the wrong approach.*^46^

### 3. Tensions in the industry use of ‘complexity’

Despite the claim that the complexity of the problem prevents simple solutions, the solutions most commonly proposed by industries do not themselves appear any more or less complex than those they reject—such as the industry emphasis on recommending more information, education and individual responsibility. In one example, Tennents’ in 2005 (then owned by brewers InBev, now under different ownership) responded to the problem of alcohol and homelessness by arguing that individuals need to take responsibility for their consumption patterns and by stating their preference for addressing the complexity through ‘partnership’ (see online supplementary file 1).^47^

Similarly, the Portman Group in a response to a European Union consultation warns of the complexity of alcohol harms.^31^ Their submission also states that ‘*health warning labels are an ineffective strategy to minimise alcohol-related harm, particularly in the ‘at-risk’ groups*’—while elsewhere the same document points to the importance of labelling and providing simple responsibility messages (eg, ‘Please enjoy responsibly’).^31^

BDSA, too, argues that obesity is a complex issue, with many contributory factors, including physical activity, diet and lifestyle, which must all be taken into account. However, the solution is simple: ‘*Providing people with a choice of beverages helps ensure they drink enough—variety of drinks choice is key’*.^48^

As with SSBs, arguments about complexity are often combined with a recommendation for educational approaches, as in this statement from McDonald’s chief executive officer (CEO), talking about childhood obesity:

*“The issue of obesity is complex and is absolutely one our society is facing, there’s no denial about that… But if you break it down I think there’s an education piece: how can we better communicate to individuals the importance of a balanced diet and taking care of themselves?”* (Steve Easterbrook, McDonalds CEO, 8 January 2008).^49^

The gambling industry also appears to favour simple solutions to the complex issue of problem gambling. ABB, for example, describes its approach to minimising gambling-related harm as based on ‘*effective education and prevention techniques, designed to prevent people getting to a stage where they have a problem with their gambling*.’^50^ However, international evidence suggests that structural changes, such as precommitment, one-dollar maximum bets or other machine design changes, may yield significantly more effective harm minimisation effects than measures focused on the individual and designed to encourage ‘responsible gambling.’^51^

We also noted that complexity arguments often appear alongside statements that de-emphasised the problem, by stating that it is in decline. Thus, the argument against the implementation of effective population-level measures appears to be constructed from at least five frequently recurring elements, which are commonly used across industries and across countries (box 1).

Box 1 **The Unhealthy Commodity Industry playbook on ‘complexity’**

**Arguments about the nature of the problem:**

1. The problem affects a minority of the population and/or is declining;
2. The aetiology is complex, so we cannot blame a single product or product category; (keywords/phrases: ‘demonisation’; ‘no single solution’; ‘no one size fits all’);
3. Consumption or use of the product makes only a small contribution to public health harms.

**The preferred solutions of unhealthy commodity industries:**

1. Arguments that taxation and other population-level measures are too simple a solution to such a complex problem; (key phrases: ‘no one-size fits all’; ‘no magic bullets’), and
2. Claims that information, education and personal responsibility are the appropriate and/or most effective solutions.

## Discussion

The concept of complexity in causation is used in very similar ways by a range of UCIs to dispute their role in causing the problems and to argue against regulatory or legislative solutions. Used in this way, complexity represents an industry ‘frame’, used to influence public perceptions and political debate.^52^ Understanding such frames can be an important part of analysing policy debate and developing and implementing effective policy. Framing has been widely used by the tobacco industry to influence discussions about tobacco and the necessity for regulation.^52 53^ This analysis shows that industry framing aims to influence a range of other policy debates, too; in the case of the alcohol industry, the focus is on minimum unit pricing of alcohol and marketing restrictions; for the SSB industry, the focus is typically on sugar tax; for the food industry, the focus is on policy solutions such as ‘fat taxes’ or sugar taxes; and for the gambling industry, it is on the tighter regulation of harmful industry products.^44^

The examples given are illustrative, and many more could be given. Overall, the framing of the problem appears intended to suggest that, because of problem complexity, we need, but do not have, perfect evidence on how to solve the problem, while current ‘simplistic’ solutions (typically, public health policies) are unacceptable. There are variations between the industries examined, and in particular, the examples from the alcohol industry appear more detailed and developed than for other industries and emerge from a wider range of responsibility organisations. This may reflect their longer history of disseminating such information, for example, through organisations such as ICAP.^54^ It may also reflect links both to the tobacco industry and to think-tanks linked to the tobacco and alcohol industries.

Arguments about the complex, multifactorial aetiology of CHD and cancer have long been used by the tobacco industry to dispute the epidemiological and other evidence.^7^ This approach to the evidence has also been documented in other industries,^52 55 56^ and the use of double standards in demands for evidence is a characteristic of many other fields.^9^ For example, car manufacturers fought the mandatory introduction of airbags and seatbelts in the 1960s as ineffective,^57^ and the alcohol industry and motoring organisations did the same with the introduction of the breathalyser to tackle drink driving in the 1960s.^58^ Demands for perfect evidence, while misrepresenting the existing evidence, can also be observed in climate change denialism.^9^

### The logical fallacies underlying industry arguments

There are numerous inconsistencies and fallacies underpinning the arguments presented by UCIs. One fallacy is that, because the aetiology of a particular problem is complex, we cannot be sure that one aspect, for example, alcohol, is causally implicated. A related fallacy is that perfect evidence is needed to establish any risk factor. Yet all health and social problems have complex causes, at least in the way that industries use the term. For example, lung cancer is a disease with a complex aetiology, but smoking is its most important risk factor: and most cases of lung cancer occur in smokers.^59^ Yet there is no ‘perfect’ evidence of lung cancer and smoking: there are no randomised controlled trials (RCTs), for obvious reasons. Even if there were, the evidence would still not be ‘perfect’ because such hypothetical RCTs would not include all current and future smokers, from all possible contexts.

The industry arguments presented in this paper imply the existence of what can perhaps be termed the ‘complexity fallacy’: that complex problems can only be addressed by complex solutions. Policymakers should be aware of this so that it may be taken into account when weighing industry arguments that ‘Nothing can be done until everything is done’. However, complexity in itself is not an argument against being able to identify significant contributing factors to a particular problem and to start addressing these. For example, smoking is a complex public health problem, with many interconnecting social, cultural and economic influences; yet, multifactorial causation does not preclude identifying and tackling one part of the problem, such as marketing.

## Conclusions

The use of the concept of complexity to counter effective interventions appears to be part of a cross-industry ‘playbook’, given the similar way it is deployed and the very similar language used. These similarities between industries are not surprising given the revolving doors between industry bodies and the interlocking directorates.^60 61^

The use of complexity is likely to be part of a wider strategy by industry bodies to promote a parallel evidence base, and thereby to influence policy and to weaken purely public-health based policies.^52^ Our analysis highlights the importance of comparative cross-industry analyses in understanding this playbook. With further data, it may be possible to identify particular arguments and combinations of arguments, put forward by different proponents, for example, industry-funded researchers, politicians or think-tanks, which may be particularly characteristic of industry involvement. The combination of phrases relating to ‘complex problem/simplistic solutions/one-size-fits-all solutions’ appears to be one of these.

Finally, it should be noted that public health problems are genuinely complex, and discussion of their complexity is of course essential for the genuine exploration of problems and solutions. However, for public health proponents, complexity provides a framework to help develop a sophisticated understanding of the problem and to determine how to intervene at different levels and across different outcomes.^62 63^ In contrast, industries often use the term loosely, corresponding to definitions of ‘complicatedness,’ rather than ‘complexity’.^63^ More importantly, ‘complexity’ seems not to be used as a starting point for further analysis or for effective intervention but apparently to distract the audience from the industry’s contribution to the problem and to promote inaction or ineffective solutions. Overall, the industry framing of complexity seems consistent with a warning by Roberts and Edwards:

*Complexity is a strategy used by professional elites to maintain control. Proclaiming that a problem is complex, is shorthand for saying that you have no role in solving it*.^64^

**What is already known on this subject**

Historically, unhealthy commodity industries have sought to manipulate the public and scientific understanding of public health problems. The history of the tobacco industry in this regard is well known, but there are also more recent examples of attempts by industry to influence the agenda. For example, the sugar and fast food industries frequently dispute the evidence that diet is a contributor to obesity, instead seeking to portray obesity as primarily a problem of ‘energy imbalance’—thus distracting from their own role in causing the problem.

**What this study adds**

This study shows how the concept of ‘complexity’ is used in this way by a range of industries—including the gambling, alcohol and food industries. Complexity therefore appears to be an important aspect of a cross-industry ‘playbook’. Researchers and policymakers should be aware of this, as use of this playbook tends to undermine effective action—in particular, to undermine effective regulation of those industry activities that are harmful to the public’s health.
