# Supplementary material for: ‘Nothing can be done until everything is done’: the use of complexity arguments by food, beverage, alcohol and gambling industries
Source: J Epidemiol Community Health. 2017 Oct 4;71(11):1078–83. doi: 10.1136/jech-2017-209710 (PMC5847098; doi:10.1136/jech-2017-209710)
Supplement: Supplementary file 1 [file jech-2017-209710supp001.pdf]

## Supplementary File 1: Additional examples of complexity arguments across industries

### (i) Sugar-sweetened beverages

Consumption of sugar-sweetened beverages (SSBs) is a risk factor for obesity, cardiovascular disease, and diabetes, and SSB taxes can reduce their consumption.[1-4] The SSB industry however argues that obesity is mainly caused by "energy imbalance" – an imbalance between calories consumed, and calories expended, with an emphasis on the need for physical activity to reduce any imbalance, rather than a reduction in calorie intake.[5] It has extensively funded research and researchers to develop this argument.[5]

Similar constructions of the argument appear in statements from other bodies, for example the Competitive Enterprise Institute in the US, which has received beverage industry funding:

> *"... Soda represents only about 9% of calories consumed by the average American ... This year, soda consumption hit a 30-year low. Yet last year obesity rates in the U.S. hit an all-time high. The roots of the obesity epidemic are deeper and more complex than a single category of products"*.[6]

Beverage industry arguments commonly contrast a "complex problem" (obesity) with "simplistic approaches" or "single solutions" (typically, taxation of SSBs), as in this example from the British Soft Drinks Association (BDSA):

> "*There's ample evidence to suggest that taxing soft drinks won't curb obesity, not least because its causes are far more complex than this simplistic approach implies. ..Trying to blame one set of products is misguided, particularly when they comprise a mere 2% of calories in the average diet."* [7]

Many similar statements are made elsewhere by the BSDA,[8] and by the representative body for the non-alcoholic beverages industry in Europe, UNESDA, such as: "*Consumer behaviour is* complex ... *The pricing of soft drinks is extremely* complex….",[9] and "*Singling out one product and taxing it will not solve the obesity crisis*".[10] Individual companies also use the argument, for example, the Britvic company:

> *"We are extremely disappointed that the government is proposing to introduce a soft drinks tax from 2018. Singling out soft drinks alone will not solve the obesity problem, given the small proportion of calories they contribute in the average diet."*[11]

Here, Coca-Cola GB pairs the argument about the complexity of the problem, with a recommendation for more information and education:

> *"Obesity is a complex public health challenge, influenced by many different factors…We believe choice and information are fundamental for consumers to make the right decisions for them and their families..."*[12]

The American Beverage Association asks **"***Does the consumption of soft drinks cause obesity problems?",* and in reply states:

> *"Obesity is a complex problem that is influenced by many factors, most importantly diet, exercise and genetics. ... The key to living a healthy lifestyle is to incorporate a balanced, healthy diet that balances calories consumed and calories burned through activity and exercise."*[13]

**(ii) Alcohol**

Documents form the alcohol industry bodies ICAP (International Center for Alcohol Policies – see: http://www.ias.org.uk/What-we-do/Publication-archive/The-Globe/Issue-1-2002/The-International-Center-for-Alcohol-Policies.aspx) and IARD (International Alliance for Responsible Drinking: see: http://www.iard.org/) use similar arguments to reject a clear role for alcohol in increasing the risk of NCDs. The IARD document "Drinking and NCDs" states that:

> "*The relationship between alcohol consumption and NCDs is complex; while harmful drinking patterns are a risk factor for several NCDs, drinking in moderation has been identified as a protective factor for others. In addition to harmful drinking patterns, a mix of individual, societal, and environmental factors are associated with, and can further contribute to, an individual's risk for developing NCDs…social determinants of health (e.g. poverty, nutritional status, access to health care, and physical and social environments) remain the most important structural drivers of NCD deaths*".[14]

Similar arguments have also been used by drinks producer Diageo in respect to young people's drinking,[15] and in the following example, by the Scotch Whisky Association (SWA) to explicitly reject population-level alcohol policies. The policy in question is probably the proposal to introduce Minimum Unit Pricing in Scotland, which the SWA challenged in the European Court of Justice:

> *"… societal issues are more complex than simply having a few drinks too many or in inappropriate circumstances. Indeed, to simply blame alcohol misses the target, perhaps because to find the true cause and solution is seen as too difficult and complex.*" (Scotch Whisky Association, 2012).[16]

The following example comes from Alcohol Beverages Australia: (See: http://www.alcoholbeveragesaustralia.org.au/information/health/)

> *"Multiple risk factors are involved in the development of health problems and the mix is different for each person, but they include genetic, environmental and behavioural variables…Alcohol, like diesel engine exhaust, air pollution, processed meat, soot, solar radiation, salted fish and wood dust, has been linked to cancers and it is generally accepted the risk increases as consumption increases".*

Other examples include:

*The issue of alcohol abuse is a complex issue, and requires a partnership approach between government, retailers and drinks makers…it's up to individuals to take responsibility for what they consume".*. Tennents/InBev spokesperson, (October 2005).[17]

Here ICAP in 2007 specifically disputes the possibility of effective policy solutions, alluding to a characteristic aspect of complexity, non-linearity:

*"A linear relationship with an intervention leading to a specific outcome is not possible. As a result, interventions that purport to offer a simple solution are only avoiding the complexity of the problem…it should be noted that the vast majority of policy measures and prevention measures are not evaluated, and that many approaches are designed or implemented in such a way to make evaluation difficult or meaningless... In reality, given the complexity of a behaviour such as drinking and the involvement of a broad range of confounding factors, establishing such a cause-and-effect relationship is rarely possible…complexity and variation in drinking should forewarn against 'off-the-peg' solutions."*[18]

In the following example, by the Chief Executive of the Scotch Whisky Association (SWA) says:

*"I never came across a policy problem that had one single simple solution. They're all complex, they all have unpredictable human elements within them, they all have unintended consequences. So I think when we're thinking about the policy response in this area you have to avoid quick fixes, you have to avoid simple solutions. They don't work, not necessarily because they're ineffective in their own right, but because they aren't the whole story and they distract attention from measures that do work... We don't need detailed legislation…".* (David Frost, Chief Executive, Scotch Whisky Association, 2015).[19]

Similarly, the submission from the European Spirits Organisation states (in very similar wording to that of the World Spirits Alliance, above):

*"The issue of alcohol related harm is complex and there is no "one-size-fits-all" approach. Members of the WHO must be able to adapt strategies to the national/local needs."*[20]

One further specific context in which the alcohol industry uses complexity is to argue against global policy recommendations akin to the World Health Organization Framework Convention for Tobacco Control (FCTC). In evidence submissions made by alcohol producers to the World Health Organisation during the development of the WHO Global Strategy to Reduce the Harmful Use of Alcohol, the Portuguese Brewers Association, the Italian Brewers Association, the Belgian Brewers Association, the German Brewers Association, the Federation of the Finnish Brewing and Soft Drinks Industries, the Austrian Brewers Association, the British Brewers Association, 'The Brewers of Europe', and 'The Worldwide Brewing Alliance' separately submitted the following identical text:

*"In our opinion, sustainable, effective strategies to reduce alcohol-related harm need to:…Consider fully the diversity of societies, cultures and tradition. From a global perspective, therefore, the most effective approach needs to reflect:… The diversity of Member States, and not imply a one-size-fits all policy prescription; The need for Member States to consider tailoring their national approaches based on what is feasible, effective and relevant to their particular economic, social and cultural contexts;".*[20]

Other alcohol industry examples frequently argue that the problem is too complex for simple solutions (or "magic bullets").[21]

**(iii) Food**

The UK Food and Drink federation reveals some inconsistencies in a letter to the Head of the NHS, where it argues against simple solutions which focus on one ingredient (sugar), while elsewhere in the same letter arguing *for* similarly simple solutions (such as nutrition labelling), and focussing on the importance of removing other single ingredients (e.g. trans fats: "we have virtually eliminated artificial trans fats in products" [22]):

> *"Obesity is a complex problem which cannot be reduced to the demonisation of one ingredient [sugar]…. …as you acknowledge, a world-leading salt reduction programme is at the forefront of these [industry] achievements … in providing nutrition information on pack".*[22]

The food industry also uses the argument to reject public health attempts to address specific dietary factors. Public health interventions, such as soda taxes, tend to target foods containing one or more of ingredients associated with poor diets – particularly high levels of fat, saturated fat, sugar, and salt (HFSS products). For example, the Director General of the Food and Drink Federation, the representative body for the UK food and drink manufacturing industry, in a 2015 letter to the head of the National Health Service (NHS) in England, makes the case why sugar should not be blamed for obesity:

> *It is not feasible to blame any one food product or beverage as being a sole contributor to obesity problems considering people consume calories from many different sources* American Beverage Association."[13]

This argument is also used by a McDonalds Vice-President:

> *"Demonising or taxing specific nutrients is not the right tool to address obesity and offers a simplistic solution to a complex problem"* Julian Hilton-Johnson, corporate vice-president, corporate relations for Europe, March 10th 2016).[23]

**(iv) Gambling**

The Association of British Bookmakers states that, '*It is not possible to clearly single out some types of gambling activities as being specifically associated with problem gambling.*'[24]

Other industries also argue that complexity in causation makes it difficult to blame specific products. For example, in a 2014 report the betting company Gala Coral state that:

*"Problem gambling is complex and is about the person not the specific product. Gala Coral, and the bookmaking sector as a whole, is determined to play a leadership role in identifying appropriate measures that improve player protection for those who need it whilst, as far as possible, protecting the freedom of the millions who enjoy betting responsibly".*[25]

**References for web material**


1 Hruby A, Manson J, Qi L, *et al.* Determinants and Consequences of Obesity. *American Journal of Public Health* 2016;**106`9**:1656-62.

2 Nakhimovsky S, Feigl A, Avila C, *et al.* Taxes on Sugar-Sweetened Beverages to Reduce Overweight and Obesity in Middle-Income Countries: A Systematic Review. *PLOS ONE* 2016;**11**:e0163358.

3 Hu F. Resolved: there is sufficient scientific evidence that decreasing sugar-sweetened beverage consumption will reduce the prevalence of obesity and obesity-related diseases. *Obes Rev* 2013;**14**:606-19.

4 Narain A, Kwok C, Mamas M. Soft drinks and sweetened beverages and the risk of cardiovascular disease and mortality: a systematic review and meta-analysis. *Int J Clin Pract* 2016;**[epub ahead of print]**:doi: 10.1111/ijcp.12841.

5 Nestle M. *Soda Politics*. NY: Oxford University Press 2015.

6 Soda taxes slim wallets, not waistlines. USA Today editorial by Michelle Minton, Available at: http://www.usatoday.com/story/opinion/2016/06/30/soda-taxes-slim-wallets-not-waistlines-bloomberg-oakland-san-francisco-philadelphia-fat-mexico-public-health-research-facts-column/86518390/?utm_source=feedblitz&utm_medium=FeedBlitzRss&utm_campaign=news-op. Accessed 8th July 2016.

7 Sugary drinks tax 'effective public health measure'. BBC News, 1st November 2013. Available at: http://www.bbc.co.uk/news/health-24759517. Accessed 8th July 2016.

8 Sugary drinks should carry health warnings. The Telegraph 28th may 2014. Availble at: http://www.telegraph.co.uk/news/health/news/10858389/Sugary-drinks-should-carry-health-warnings.html. Accessed 8th July 2016.

9 Why Food & Drink Taxes Won't Work: The facts about food and drink taxes. Available at: http://www.fooddrinktax.eu/. Accessed 8th July 2016.

10 Soft drinks vendetta called into question by obesity study. *The Grocer 22nd April 2016*.

11 Coca-Cola and Britvic hit back at sugar tax for 'singling out soft drinks'. Marketing Week, March 17th 2016. Available at: http://www.marketingweek.com/2016/03/17/coca-cola-and-britvic-hit-back-at-extremely-disappointing-sugar-tax/. Accessed 8th July 2016.

12 Tackling Obesity: Choice and Information. Coca-Cola Website, 24th March 2015. Available at: http://www.coca-cola.co.uk/stories/health/choice-and-information/tackling-obesity-choice-and-information. Accessed 8th July 2016.

13 American Beverage Association. Questions & Answers: Soft Drinks and Obesity. http://www.ameribev.org/nutrition-science/obesity/qas/ Accessed 8th July 2016.

14 Drinking and NCDs. IARD (International ALliance for |Responsible Drinking) Health Review. Available at: http://www.iard.org/wp-content/uploads/2016/06/HR-NCDs.pdf. Accessed 26th July 2016.

15 Diageo Australia's submission to the standing committee on social issues inquiry into strategies to reduce alcohol abuse among young people in New South Wales, 2013. Available at:

https://www.parliament.nsw.gov.au/committees/DBAssets/InquiryReport/ReportAcrobat/5863/Final%20Report%20-%20Strategies%20to%20reduce%20alcohol%20abuse%20.pdf. Accessed 14th August 2016.
16 Matured to be enjoyed responsibly. Scotch Whisky Association, 2012, 3rd Edition.. Available at: http://www.scotch-whisky.org.uk/media/44727/mtber_web_jul24.pdf. Accessed 14th August 2016.
17 UK: Super strength drinks under attack, Just-Drinks.com 21st Oct 2005.
18 Stimson G, Grant M, Choquet M, *et al. Drinking in context: Patterns, interventions and partnerships*. New York: Routledge 2007.
19 Presentation by David Frost, Chief Executive, Scotch Whisky Association; Westminster Social Policy Forum, 17th March 2015.
20 WHO Public Hearing on the Harmful Use of Alcohol, Volume IV, Geneva, 2009. Available at: http://www.who.int/substance_abuse/activities/4ai.pdf?ua=1. Accessed 14/11/16.
21 UK: PM warns drinks industry to up anti-binge drinking presence. Just-Drinks.com 21st Nov 2007.
22 Food and Drink Federation open letter to Simon Stevens, Chief Executive of NHS England, 3rd June 2015. Available at: https://www.fdf.org.uk/news.aspx?article=7261. Accessed 15th May 2017.
23 McDonald's European comms chief on reputation, obesity, taxes - and whether Ronald McDonald is retired. PR Week, March 10, 2016. Available at: http://www.prweek.com/article/1386686/mcdonalds-european-comms-chief-reputation-obesity-taxes-whether-ronald-mcdonald-retired. Accessed 8th July 2016.
24 Association of British Bookmakers (ABB). The truth about betting shops and gaming machines. ABB submission to Department of Culture, Media and Sport Triennial Review, April 2013, p. 3. Available at: https://www.gov.uk/government/uploads/system/uploads/attachment_data/file/248922/Association_of_British_Bookmakers.pdf. Accessed 13th November 2016.
25 Gala Coral Group: Results for the 12 weeks ended 12 April 2014. Available at: http://www.galacoral.co.uk/~/media/Files/G/Gala-Coral/reports-and-presentations/quarterly-report/q2-fy14-press-release-final.pdf. Accessed 13th November 2016.
